# An extended logistic model of photodynamic inactivation for various levels of irradiance using the example of *Streptococcus agalactiae*

**DOI:** 10.1038/s41598-020-71033-7

**Published:** 2020-08-25

**Authors:** Michal Brasel, Michal Pieranski, Mariusz Grinholc

**Affiliations:** 1 Faculty of Electrical Engineering, West Pomeranian University of Technology, 26 Kwietnia 10, 71-126 Szczecin, Poland; 2 Intercollegiate Faculty of Biotechnology, Laboratory of Molecular Diagnostics, University of Gdansk and Medical University of Gdansk, Abrahama 58, 80-307 Gdańsk, Poland

**Keywords:** Computational models, Computational biology and bioinformatics, Microbiology

## Abstract

Irradiance is an important factor influencing the acceleration of microorganism mortality in photodynamic inactivation (PDI) processes. Experimental observations of PDI processes indicate that the greater the irradiation power is, the faster the decrease in the population size of microorganisms. However, commonly used mathematical models of PDI processes usually refer only to specific values of irradiance without taking into account the influence of change in irradiance on the dynamic properties of inactivation. The main goal of this paper is to analyze the effect of irradiance on the PDI process and attempt to mathematically model the obtained dependencies. The analysis was carried out using the example of photodynamic inactivation of the bacterium *Streptococcus agalactiae* with the adopted Logistic PDI model optimized for several selected levels of irradiance. To take into account the impact of changes in irradiation power on the PDI model, the selected parameters were made appropriately dependent on this factor. The paper presents several variants of parameter modification with an evaluation of the model fitting quality criterion. The discussion on appropriate selection of parameters to be modified was carried out as a comparative analysis of several case studies. The extended logistic PDI model obtained in the conducted research effectively describes the dynamics of microorganism mortality in the whole tested irradiation power range.

## Introduction

This manuscript presents the results of experimental research on the photodynamic inactivation (PDI) process using the bacterium *Streptococcus agalactiae* as an example. Group B Streptococcus (GBS) bacteria, the most common species of which is *S. agalactiae*, are Gram-positive pathogens representing one of the major causes of life-threatening bacterial infections in newborns and infants. Perinatal infections can occur in the form of early-onset disease (EOD) or late-onset disease (LOD). Early GBS infection in newborns occurs at up to 7 days of life, causing sepsis, pneumonia or meningitis, while the late form of infection occurs between the 7th and 89th days of life, causing septicemia or meningitis, as well as inflammation of the respiratory system, joints and connective tissue^1^. In recent years, an alternative form of prevention of perinatal infection has been proposed. Intensive ongoing work is focused on the generation of an effective vaccine directed against polysaccharide capsular and protein surface antigens of *S. agalactiae*. Nevertheless, the occurrence of several serotypes of this bacterium and the high percentage of strains intractable to serotyping methods significantly impede the development of a universal vaccine. Therefore, the ideal solution for the prevention of perinatal GBS infections seems to be the design of a safe and fast treatment for eradication of group B streptococci, which would be administered to all pregnant women immediately before giving birth (at the beginning of the regular systolic or shortly after rupture of the fetal membranes), regardless of the outcome of the screening test. The bacterial eradication approach, which, for many years, has been the primary subject of our research using both in vitro and in vivo studies, is PDI^2–5^. PDI causes damage to microbial cells by generating reactive oxygen species (ROS) induced by the interaction between visible light of an appropriate wavelength and a light-sensitive chemical called a photosensitizer (PS). PDI involves the absorption of a photon of light leading to excitation of the PS to its short-lived excited singlet electronic state. This singlet-state PS can undergo an electronic transition to a much longer-lived triplet state. The longer lifetime allows the triplet PS to react with ambient (ground state) oxygen by one of two different photochemical pathways, called Type 1 and Type 2 mechanisms. Type 1 involves an electron transfer to produce superoxide radical and then hydroxyl radicals (HO^·^), while Type 2 involves energy transfer to produce excited state singlet oxygen (^1^O_2_). The generated ROS, such as singlet oxygen, can exert cytotoxic effects against a variety of biomolecules, e.g., proteins, lipids, cell membranes or genetic material^6^.

The PDI outcome depends on numerous factors. Irradiance is one of the most important factors influencing the acceleration of microorganism mortality in PDI processes. Experimental PDI tests are most often performed with a constant irradiance for a specified period of time^7–12^. Therefore, commonly used mathematical models of PDI processes usually refer only to specific values of irradiance without taking into account the influence of changes in irradiance on the dynamic properties of inactivation. In photodynamic therapy (PDT), the permissible range of irradiance may be limited for various reasons; thus, the choice of specific irradiation level may be limited. This is important because various levels of irradiation power correspond to various irradiation times required to achieve the same inactivation result. Therefore, estimation of the required irradiation time for various levels of irradiation power may be very helpful. To predict the dynamic properties of the PDI process for various levels of irradiation power, a properly extended PDI model is needed. Therefore, the present study was focused on analyzing the effect of irradiance on the PDI process and attempted to mathematically model the obtained dependencies.

The manuscript presents the results of experimental research on PDI for four selected levels of irradiance: *µ*_1_ = 70 mW/cm^2^, *µ*_2_ = 52.5 mW/cm^2^, *µ*_3_ = 35 mW/cm^2^, and *µ*_4_ = 17.5 mW/cm^2^, corresponding to 100, 75, 50 and 25% of the maximal output power produced by the LED, respectively. The commonly known Logistic PDI model was used to approximate the obtained results^13–17^. The parameters of this model were fitted to the collected experimental data by using the root mean square error (RMSE) minimization method. Based on the conducted research, it was found that the level of irradiance significantly affects the dynamic properties of the inactivation process of microorganisms. In particular, it has been observed that the greater the radiation power is, the faster the decline in bacterial population. For this reason, the parameters of the selected PDI model optimized for various irradiation levels are no longer the same. This indicates that there is a need to take into account the irradiance in the PDI model used. The main goal of this manuscript is to analyze the effect of the irradiance on the dynamics of the inactivation process using the example of the bacterium *S. agalactiae* and to propose the inclusion of this effect in the Logistic model by using a parameter variation approach. Because the logistic model has as many as three parameters, it may be a complicated task to modify these parameters appropriately. The following questions arise: should all model parameters be changed, and if not, which ones should be changed and how? A useful method in the selection of parameters that are most suitable for modification may be to examine their sensitivity to changes in the irradiance level. This approach is proposed in the manuscript. First, the effect of irradiation level on the survival curves of the Logistic model is described in the manuscript. Then, the issue of including obtained dependencies in the considered model is investigated. The main results of the experimental research on PDI are presented as several case studies for alternative variants of Logistic model modification, i.e., for the following cases: A, for all modified parameters; B, for two modified parameters; C and D, for one modified parameter; and E, for all constant parameters. The discussion on appropriate selection of parameters to be modified is described as a comparative analysis of the considered cases. The fitting quality and the degree of complexity of the proposed modified models were the most important comparative criteria utilized in this analysis. The extended Logistic PDI model obtained from these studies effectively describes the dynamics of microorganism mortality in the whole tested range of irradiance.

## Materials and methods

### Bacterial strains and reagents

This study was conducted with the *S. agalactiae* strain ATTC 27,956 isolated from an infected bovine udder, representing Lancefield's group B streptococci. Bacteria were grown in tryptic soy broth (TSB) (Biomerieux, France). A stock solution of rose bengal (RB) (Sigma-Aldrich, Germany) was prepared in sterile double-distilled water and kept at 4 °C. All solvents and other chemicals were of analytical grade.

### Light source

The LED light source with an emission maximum at 515 nm (FWHM = 33 nm) was custom made by EMD Technology (Warsaw, Poland). The light source was precisely characterized in our previous publication^18^.

### Inactivation experiment

*Streptococcus agalactiae* was grown overnight (16–20 h) in glass tubes containing 5 ml of TSB medium with shaking (150 RPM) at 37 °C. The culture was adjusted to an optical density of 2.4 McFarland (8 × 10^7^ CFU/ml) units in fresh TSB medium. A 2 µM solution of RB was prepared from the stock solution and then diluted 10× with bacterial culture to obtain a final 0.2 µM concentration of RB. Samples were incubated in the dark for 15 min at 37 °C with shaking and then centrifuged twice and washed with PBS. Then, 100-µl bacterial cultures in PBS were transferred into 96-well plates and irradiated for 0, 1, 2, 5, 10, 15, 20, 30, 45, 60, 120, 180, 240, 300, or 360 s. Irradiated samples were serially diluted in PBS, plated onto Columbia blood agar plates and incubated at 37 °C. After 24 h of incubation, colony forming units (CFU) were counted. Experiments were performed in three independent repetitions for four selected levels of irradiance: *µ*_1_ = 70 mW/cm^2^, *µ*_2_ = 52.5 mW/cm^2^, *µ*_3_ = 35 mW/cm^2^, *µ*_4_ = 17.5 mW/cm^2^, corresponding to 100, 75, 50 and 25% of the maximal output power produced by the LED, respectively. In the course of inactivation experiment, the temperature of PDI treated bacterial culture was measured using waterproof pocket-size pH-meter CP-105 for pH, mV, redox potential and temperature measurement (range: − 5/60 °C, accuracy: ± 0.8 °C) (Elmetron, Poland).

### Rose bengal uptake

Microbial OV cultures were adjusted to an optical density of 2.4 McFarland units in TSB medium. Bacterial suspensions were centrifuged and resuspended in PBS and then mixed with RB to obtain a final concentration of 0.2 µM. Samples were incubated for 15 min at 37 °C and immediately centrifuged at 10,000*g* for 3 min after incubation. Supernatants (1 ml) were then transferred into BRAND UV cuvettes (optical path length 1 cm), and the absorbance at 549 nm was measured by a SPECORD PLUS spectrophotometer (Analytic Jena). Additionally, each sample was serially diluted end plated for CFU enumeration. Using the molar extinction coefficient (ε = 95,000), the number of RB particles absorbed by a single bacterial cell was calculated according to the formula below^19,20^:

$$ n = \frac{{\left[ {A_{0} - A_{t} } \right] \times N_{A} }}{\varepsilon \times l \times N} $$

in which the variables are defined as follows: *n* is the number of RB molecules per bacterial cell, *A*_0_ is the initial absorbance of bacterial supernatant administered with RB (blanked with the supernatant of bacterial culture not administered with RB), *A*_t_ is the absorbance of supernatant of bacterial culture administered with RB for 15 min (blanked with the supernatant of bacterial culture not administered with RB), *N*_*A*_ is Avogadro’s Constant (6.02214086 × 10^23^/mol), ε is molar extinction coefficient (M/cm), *l* is optical path length (cm), *N* is number of bacterial cells (CFU/L).

### Ethical approval

The manuscript contains no data concerning animal studies, studies involving human subjects or inclusion of identifiable human data or clinical trials; thus, no ethical approval was required.

## Results

### Rationale and control experiments supporting experimental conditions

**Figure 1:**
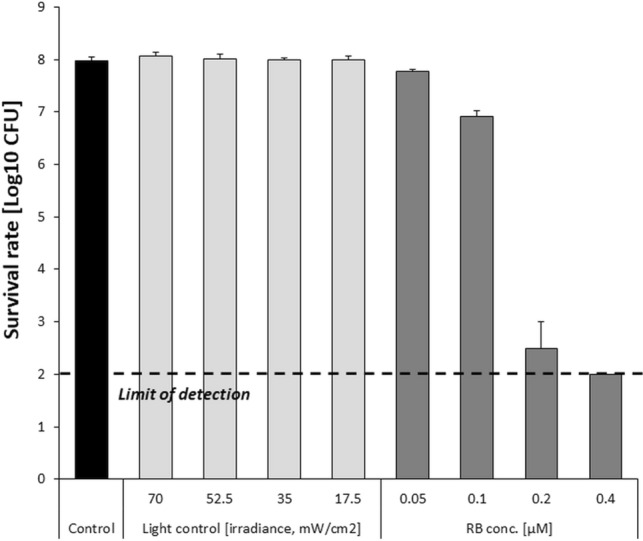
Dose-dependent PDI efficacy against *S. agalactiae*. Microbial overnight cultures (8 × 10^7^ CFU/ml) were treated with up to 0.4 µM RB and exposed to a light dose of 20 J/cm^2^ (λ_max_ 515 nm). After illumination, samples were serially diluted, streaked horizontally, and incubated at 37 °C for 24 h, and then colonies were counted. Control groups included: (1) cells that were not treated with PSs or light (marked as “control”); (2) cells treated with light for 6 min with various irradiance and no PS administration (marked as “light control”). The detection limit was 100 CFU/ml. The values are the means of three separate experiments. Error bars represent standard deviations (SDs).

To ensure adequate computational analysis, RB concentration was adjusted to result in moderate bacterial killing (not exceeding the detection limit; reduction by maximum 5 log_10_ units in viable counts) when excited with visible light within few minutes. Employing higher PS concentrations reached microbial eradication regardless irradiance and prevented adequate modeling (Fig. 1). RB concentration of 0.2 µM was considered optimal as exerted the most effective bacterial killing above detection limit (Fig. 1).

**Figure 2:**
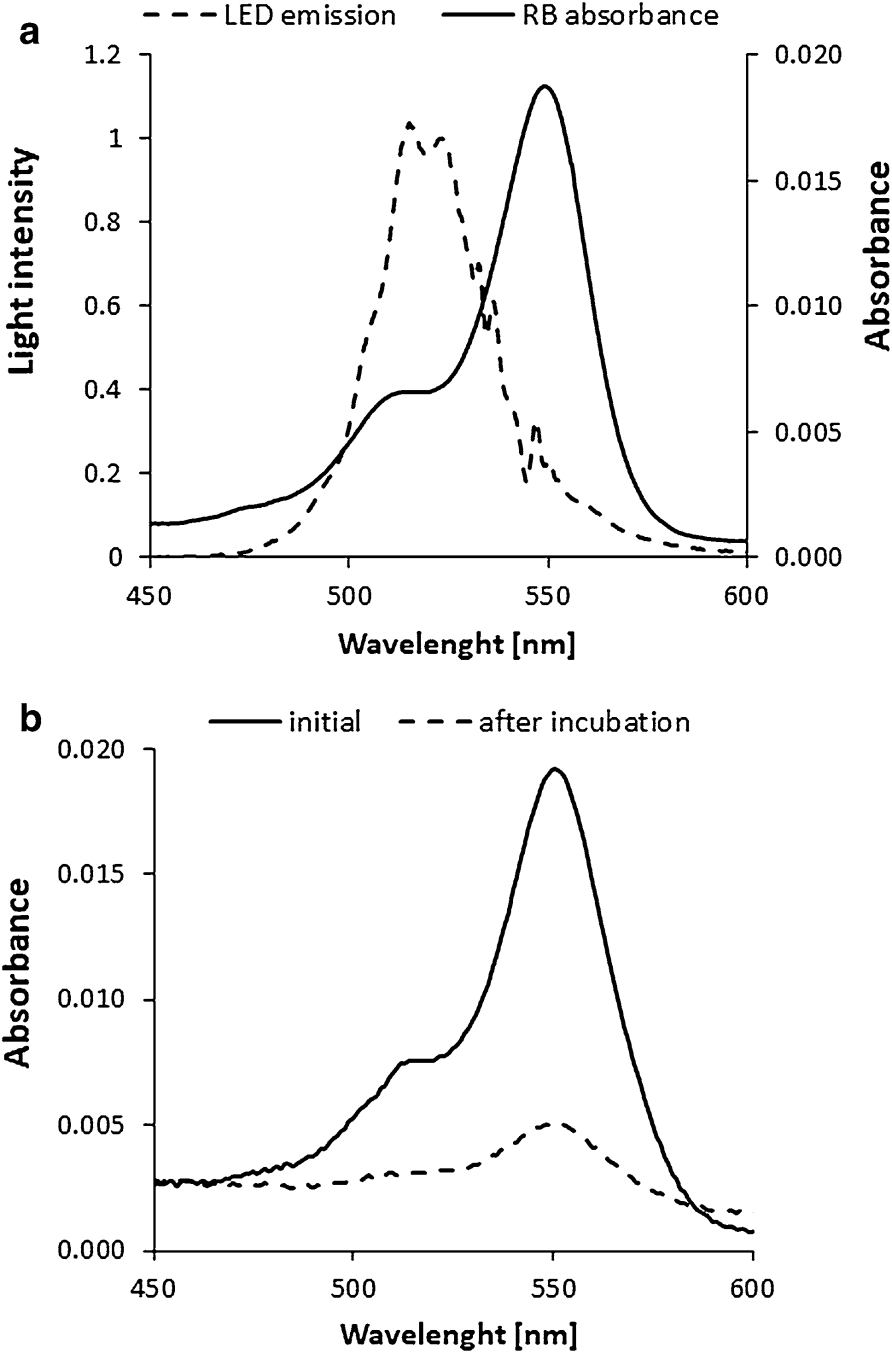
LED characterization and RB absorption spectra. (**a**) The emission spectrum of the LED is presented, and the maximum intensity is observed at 515 nm. A rose bengal (0.2 µM) absorption spectrum is presented in the wavelength range of interest. (**b**) The absorbance spectra of bacterial culture supernatant administered with 0.2 µM RB (termed “initial”) and supernatant of bacterial culture administered with RB for 15 min and centrifuged (termed “after incubation”) are presented.

Employed LED light source effectively excites studied PS by overlapping its absorption spectrum (Fig. 2a). Though negatively charged in water solutions, RB revealed substantial microbial uptake (9.56E + 05) leading to photodynamic inactivation of *S. agalactiae* upon light treatment. The change in the absorbance spectrum of bacterial culture supernatant after 15 min incubation with RB in comparison to initial absorbance spectrum demonstrates RB uptake (Fig. 2b). The uptake of RB was calculated with the molar extinction coefficient and expressed as the number of photosensitizer molecules per bacterial cell.

Furthermore, to evidence that the observed inactivation efficacy of PDI may only be attributed to photosensitized reaction mediated by RB, the control experiments concerning light treatment with no RB administration together with temperature detection were performed. Obtained results demonstrated that light alone exerts no bactericidal activity (Fig. 1) and in the course of photodynamic treatment no substantial increase in temperature that could might affect bacterial viability was observed (Table 1).

**Table 1:** Temperature control upon light treatment using various levels of irradiance *µ* [%].

Irradiance	Temperature^a^ (°C)
*µ*_1_ = 100%	27.3 ± 0.15
*µ*_2_ = 75%	26.0 ± 0.11
*µ*_3_ = 50%	24.5 ± 0.11
*µ*_4_ = 25%	23.6 ± 0.10
Control (no light)	23.7 ± 0.10

^a^Temperature measurement was performed upon light treatment for maximum time used for modeling (6 min).

### Effect of irradiation level on the survival curves of the logistic PDI model

The PDI processes of microorganisms are most commonly modeled as microbial survival curves showing a change in the population at the time of irradiation. The population size is most often expressed as a logarithmic ratio of the current number of microorganisms (N) to the initial number (No) and is presented on semilogarithmic plots. One of the methods of describing the sigmoidal behavior of microbial survival curves is the Logistic model^21–24^. There are many variations and modifications of this model^14,25–30^. In this manuscript, the Logistic PDI model^13–17^ was chosen to analyze the dynamic properties of the inactivation process of microorganisms.

The model under consideration is the Logistic model, expressed as follows (Eq. 1):

1 $$ \log \left( {\frac{N\left( t \right)}{{N_{o} }}} \right) = N_{r} \left( {1 - \frac{1}{{1 + \left( {\frac{t}{\tau }} \right)^{p} }}} \right) $$

in which the variables are defined as follows: *N* is the number of microorganisms at irradiation time t, *N*_o_ is the initial number of microorganisms at time t = 0, *N*_r_ is parameter that describes the number of resistant microorganisms, *P* is parameter that describes the length of the curve shoulder, *τ* is parameter that describes the suddenness of the reduction in the population of microorganisms.

**Figure 3:**
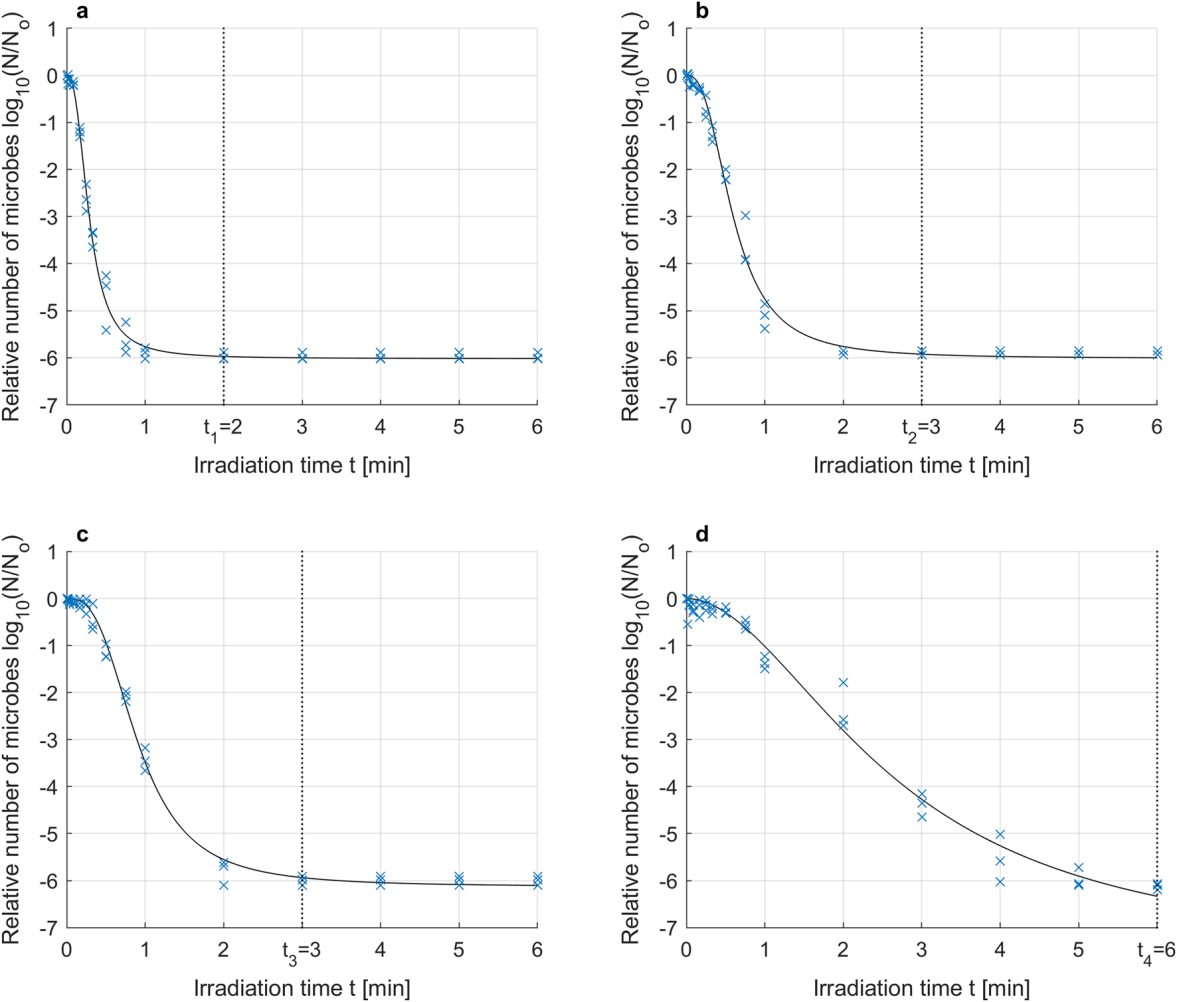
Photodynamic inactivation of *S. agalactiae* for irradiance *µ*_1_ = 70 mW/cm^2^ (**a**), *µ*_2_ = 52.5 mW/cm^2^ (**b**), *µ*_3_ = 35 mW/cm^2^ (**c**), *µ*_4_ = 17.5 mW/cm^2^ (**d**); experimental data (x) and microbial survival curve (–) of the Logistic PDI model optimized without data reduction.

Based on the collected experimental data for each of the tested irradiation levels, optimal parameters in terms of RMSE (Root Mean Square Error) were determined for the Logistic model (1). Figure 3 show the collected data along with the approximate microbial survival curves for the optimized parameters.

The values of the optimized parameters of the Logistic model and the corresponding values of the RMSE quality indicators are presented in Table 2, with RMSE calculated according to the formula (Eq. 2):

2 $$ RMSE = \sqrt {\frac{{\sum\nolimits_{i = 1}^{N} {\left( {y\left( i \right) - y_{m} \left( i \right)} \right)^{2} } }}{N}} , $$

where *y*(*i*) are the experimental data and *y*_m_(*i*) are the predicted data.

**Table 2:** Results of parameter optimization of the Logistic PDI model without data reduction for various levels of irradiance *µ* [%].

Irradiance	RMSE-optimized parameters			RMSE
	N_ropt_	τ_opt_	p_opt_	
*µ*_1_ = 100%	− 6.018	0.288	2.509	0.1923
*µ*_2_ = 75%	− 6.016	0.598	2.582	0.2144
*µ*_3_ = 50%	− 6.130	0.908	2.866	0.1630
*µ*_4_ = 25%	− 7.665	2.660	1.918	0.2757

**Figure 4:**
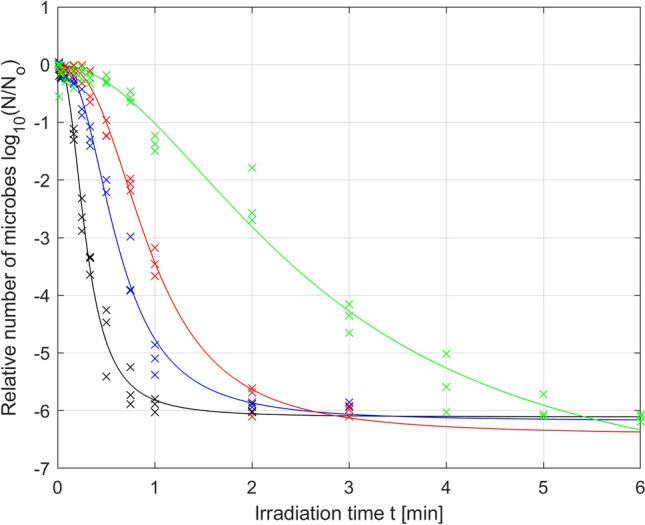
Photodynamic inactivation of *S. agalactiae* for various levels of irradiance: *µ*_1_ = 70 mW/cm^2^ (black), *µ*_2_ = 52.5 mW/cm^2^ (blue), *µ*_3_ = 35 mW/cm^2^ (red), *µ*_4_ = 17.5 mW/cm^2^ (green); experimental data (x) and microbial survival curves (–) of the Logistic PDI model optimized with data reduction.

Analysis of the results presented in Fig. 3 shows that the greater the irradiation power is, the faster the increase in microbial mortality. The change in the mortality dynamics caused by the change in irradiation power leads to the mortality curves reaching the detection limit at various irradiation times. The higher the irradiation power is, the faster the detection limit is reached, which in our case is close to log (N/No) = − 6. The individual times for reaching the detection limit for a series of irradiation power levels, namely, *µ*_1_, *µ*_2_, *µ*_3_, and *µ*_4_, are *t*_1_ = 2 min, *t*_2_ = 3 min, *t*_3_ = 3 min, *t*_4_ = 6 min, respectively, as shown in Fig. 3. After these times are exceeded, we do not truly know what happens to the population number of microorganisms. We can assume that the number of microorganisms decreases further with increasing irradiation time (to the value of *N*r), but we cannot experimentally verify this assumption. Therefore, it should be assumed that the collected experimental data marked in Fig. 3 for times greater than *t*_1_ = 2 min, *t*_2_ = 3 min, *t*_3_ = 3 min, *t*_4_ = 6 min, respectively, distort the reality to a certain degree. For this reason, the approach in which all the collected data are taken into account to optimize model parameters (also those for times after the detection limit has been exceeded) can be misleading and give nonoptimal results; therefore, model parameters should not be optimized based on these data. This is why further analysis reduced the experimental data used for optimization, using only the data collected for the first time the detection limit was reached by three independent samples. This reduction in the data collected was conducted for the irradiation powers *µ*_1_, *µ*_2_ and *µ*_3_, but for the power *µ*_4,_ this reduction was not necessary. The results of parameter optimization for the Logistic PDI model (Eq. 1) obtained based on the reduced data are shown in Fig. 4 and the corresponding Table 3.

**Table 3:** Results of parameter optimization of the Logistic PDI model with data reduction for various levels of irradiance *µ* [%].

Irradiance	RMSE-optimized parameters			RMSE
	N_ropt_	τ_opt_	p_opt_	
*µ*_1_ = 100%	− 6.112	0.293	2.426	0.2177
*µ*_2_ = 75%	− 6.182	0.615	2.478	0.2297
*µ*_3_ = 50%	− 6.418	0.950	2.645	0.1596
*µ*_4_ = 25%	− 7.665	2.660	1.918	0.2757
RMSE-weighted mean	− 6.529	1.042	2.413	0.2207
Coefficient of variation	11.18%	101.75%	13.18%	–

By comparing the results from Tables 2 and 3, it can be stated that in the case of the reduced data, the fitting quality indicators for the powers *µ*_1_ and *µ*_2_ are slightly worse than those in the case of the unreduced data, but this is not a significant deterioration, as seen by comparing Fig. 3 with Fig. 4. For the reasons described above, the parameters optimized for the reduced data should be considered more reliable. More importantly, the data reduction results in a decrease in the optimal value of parameter *N*_r_ (parameters *N*_r_ in Table 3 are smaller than the corresponding parameters in Table 2 for all irradiances). This is consistent with expectations because the optimal value of the parameter *N*_r_ describing the number of resistant microorganisms is expected to be below the detection limit. The use of unreduced data to optimize the *N*_r_ parameter makes the value of this parameter very close to the detection level, which should be considered an abnormality.

### Taking into account the effect of changes in irradiance in the Logistic PDI model

As the irradiation power changes, the dynamic properties of the photoinactivation process change, which entails changes in the optimal parameters of the identified model (Table 3). To account for these dependencies in the model, it was proposed that the parameters be changed depending on the irradiation power level. The general form of the extended Logistic model that takes into account changes in the irradiation power can be presented as follows (Eq. 3):

3 $$ \log \left( {\frac{N\left( t \right)}{{N_{o} }}} \right) = N_{r} \left( \mu \right)\left( {1 - \frac{1}{{1 + \left( {\frac{t}{\tau \left( \mu \right)}} \right)^{p\left( \mu \right)} }}} \right) $$

where *N*_r_(*µ*), *τ*(*µ*), and *p*(*µ*) are the parameters of the Logistic PDI model described by the appropriate irradiation power functions, and *μ* is the irradiation power.

The first proposed variation to take into account the effect of changes in irradiation power in the considered model is to properly vary all parameters depending on the irradiation power level (case A). However, to obtain appropriate submodels of parameter changes, it is necessary to analyze the variation in RMSE-optimized parameters depending on the change in irradiation power and approximate the collected data with appropriate parameter variation curves.

#### Case A: variation in all parameters of the Logistic PDI model

**Figure 5:**
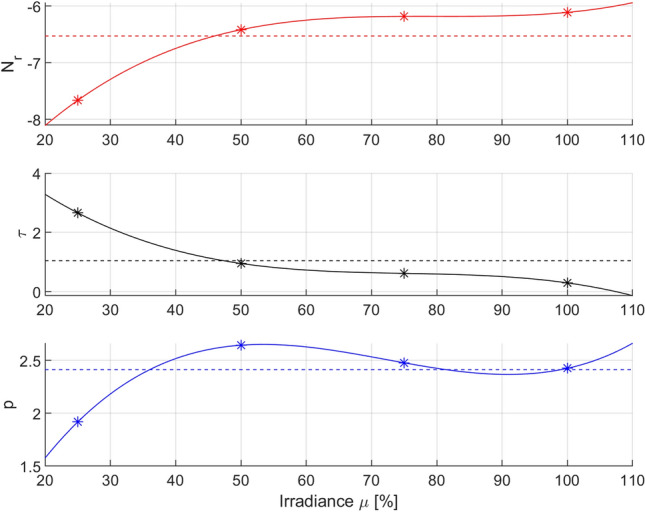
Optimal parameters of the Logistic PDI model (*) for various levels of irradiance *µ* [%] and their polynomial interpolation (–) with RMSE-weighted mean values (–). Optimization was carried out for variations in the *N*_r_, *p* and *τ* parameters.

Based on the data presented in Table 3, it is possible to analyze the variation in optimal parameters of the Logistic model and to find appropriate functions by approximating the changes in their values in the examined range of irradiation powers. In the analyzed case, the third-order polynomial functions well enough. Figure 5 shows the optimal parameters of the Logistic for the tested irradiation powers and the curves approximating the parameters accordingly.

The obtained functions describing the variation in the parameters of the Logistic model (Eq. 3) in the domain of irradiation power can be presented in the following analytical form:

4 $$ \begin{gathered} N_{rA} \left( \mu \right) = {0}{\text{.0009}} \cdot 10^{ - 2} \mu^{3} - {0}{\text{.2161}} \cdot 10^{ - 2} \mu^{2} + {17}{\text{.2507}} \cdot 10^{ - 2} \mu - {1076}{\text{.8000}} \cdot 10^{ - 2} \hfill \\ \tau_{A} \left( \mu \right) = - {0}{\text{.0015}} \cdot 10^{ - 2} \mu^{3} + {0}{\text{.3279}} \cdot 10^{ - 2} \mu^{2} - {25}{\text{.0780}} \cdot 10^{ - 2} \mu + {710}{\text{.7000}} \cdot 10^{ - 2} \hfill \\ p_{A} \left( \mu \right) = {0}{\text{.0011}} \cdot 10^{ - 2} \mu^{3} - {0}{\text{.2330}} \cdot 10^{ - 2} \mu^{2} + {15}{\text{.6713}} \cdot 10^{ - 2} \mu - {71}{\text{.2000}} \cdot 10^{ - 2} . \hfill \\ \end{gathered} $$

Equations (3) and (4) constitute one of the possible versions of the extended PDI Logistic model obtained when all the parameters are varied (case A), as indicated by the index A used in the parameter subscripts. The total number of model parameters equals n = 12 in this case, while the mean value of the RMSE quality criterion is equal to 0.2207.

The basic disadvantage of the approach in which all parameters of the identified model are modified is the increasing degree of complexity of the model with the increase in the number of modified parameters—the more parameters the model has, the more the number of submodels that describe the changes in the parameters. It is worth noting that for each varied optimal parameter, there are several coefficients, the amounts of which depend on the approximating function (in this case, on the degree of polynomials used). Moreover, this approach does not take into account differences in the sensitivity of individual parameters to changes in the irradiance or the physical significance of the parameters to be modified. However, most often, there is a situation in which individual parameters show different sensitivity to changes in irradiation power. In such cases, the variation in the less sensitive parameters may be limited or omitted without a significant deterioration in modeling quality.

An alternative approach to taking into account the changes in irradiance in the PDI model is to modify only selected parameters of the identified model. Namely, it can be assumed that only those parameters are modified that significantly react to a change in the irradiance. For this purpose, an appropriate measure of the significance of parameters of the identified model should be determined, that is, how large are their deviations from a certain characteristic value in the considered range of irradiation power. This manuscript proposes the expression of this characteristic value as a weighted mean of parameters optimized for the tested irradiation power, with weights being the inverse of the corresponding RMSE fitting quality indicators, so that optimal parameters that gave a better fit in terms of the RMSE indicator were given proportionally greater weightage for calculating the mean value. The RMSE-weighted mean values of optimal parameters calculated from the formula (Eq. 5):

5 $$ \overline{x} = \frac{{\sum\nolimits_{i = 1}^{n} {\frac{1}{{RMSE_{i} }}x_{i} } }}{{\sum\nolimits_{i = 1}^{n} {\frac{1}{{RMSE_{i} }}} }} $$

were as follows:

6 $$ \overline{N}_{rA} = - 6.529,\begin{array}{*{20}c} {} & {} \\ \end{array} \overline{\tau }_{A} = 1.042,\begin{array}{*{20}c} {} & {} \\ \end{array} \overline{p}_{A} = 2.413. $$

These values are marked with dashed lines in Fig. 5 for each parameter. Relative deviations of the optimal parameter values from their mean values (6) within the tested irradiation power range were calculated as coefficients of variation (Eq. 7):

7 $$ CV_{x} = \frac{{\sigma_{x} }}{{\left| {\overline{x}} \right|}}100\% , $$

where σ_x_ is the standard deviation:

8 $$ \sigma_{x} = \sqrt {\frac{{\sum\nolimits_{i = 1}^{n} {\left( {x_{i} - \overline{x}} \right)^{2} } }}{n - 1}} $$

The coefficients of variation of the Logistic model parameters in case A are as follows:

9 $$ CV_{{N_{rA} }} = 11.18\% ,\begin{array}{*{20}c} {} & {} \\ \end{array} CV_{\tau A} = 101.75\% ,\begin{array}{*{20}c} {} & {} \\ \end{array} CV_{pA} = 13.18\% . $$

Based on the estimated coefficients of variation, it can be concluded that parameter *N*_r_ is the least sensitive to changes in irradiation. A comparable value of variation is also shown by the *p* parameter, while the *τ* parameter is clearly more sensitive to changes in irradiation power than other parameters.

#### Case B: variation in parameters *p* and *τ* for constant *N*_r_

**Figure 6:**
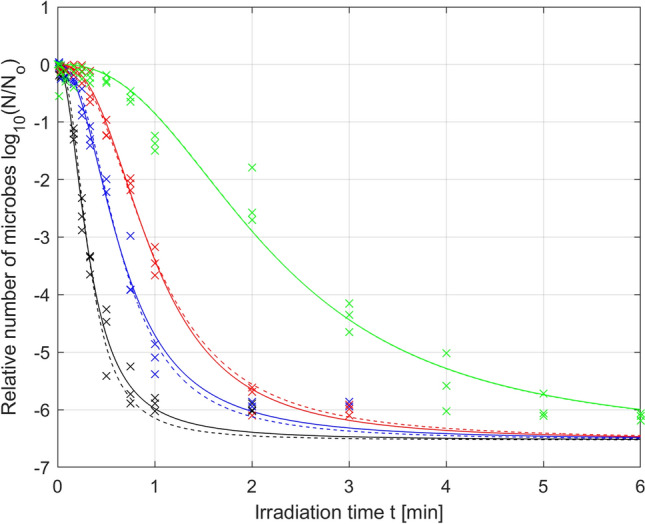
Photodynamic inactivation of *S. agalactiae* for various levels of irradiance: *µ*_1_ = 70 mW/cm^2^ (black), *µ*_2_ = 52.5 mW/cm^2^ (blue), *µ*_3_ = 35 mW/cm^2^ (red), *µ*_4_ = 17.5 mW/cm^2^ (green); experimental data (x) and microbial survival curves of the Logistic PDI model optimized for variation in the *p* and *τ* parameters (–) and only the *τ* parameter (–).

The results of the conducted analysis of parameter variation led to the formulation of a hypothesis that in the case under study, limiting or omitting the modification of parameter *N*_r_ (and possibly *p*) in the Logistic model, could give similar results in terms of the RMSE optimization to the results obtained for case A. Therefore, in the next example, it was assumed that parameters *p* and *τ* in the Logistic model will be modified, while the *N*_r_ parameter will set at a constant value equal to − 6,529, determined as (6) in case A. Of course, setting of the parameter *N*_r_ at a constant value required repeating the parameter optimization procedure for the same experimental data. The results of optimization of the *p* and *τ* parameters of the Logistic model at a constant value of the parameter *N*_r_ are shown in Fig. 6 (solid lines) and Table 4.

**Table 4:** Results of parameter optimization of the Logistic PDI model with a constant *N*_r_ parameter for various levels of irradiance *µ* [%].

Irradiance	RMSE-optimized parameters			RMSE
	N_ropt_	τ_opt_	p_opt_	
*µ*_1_ = 100%	− 6.529	0.318	2.084	0.2539
*µ*_2_ = 75%		0.655	2.230	0.2515
*µ*_3_ = 50%		0.969	2.555	0.1612
*µ*_4_ = 25%		2.198	2.410	0.3072
RMSE-weighted mean	− 6.529	1.045	2.421	0.2435
Coefficient of variation	0%	83.90%	8.87%	–

In contrast to Fig. 4, Fig. 6 shows that setting parameter *N*_r_ at a constant value with increasing irradiation time led to the convergence of all microbial mortality curves to the same fixed residual value, which was consistent with the physical significance of this parameter characterizing the number of resistant microorganisms.

**Figure 7:**
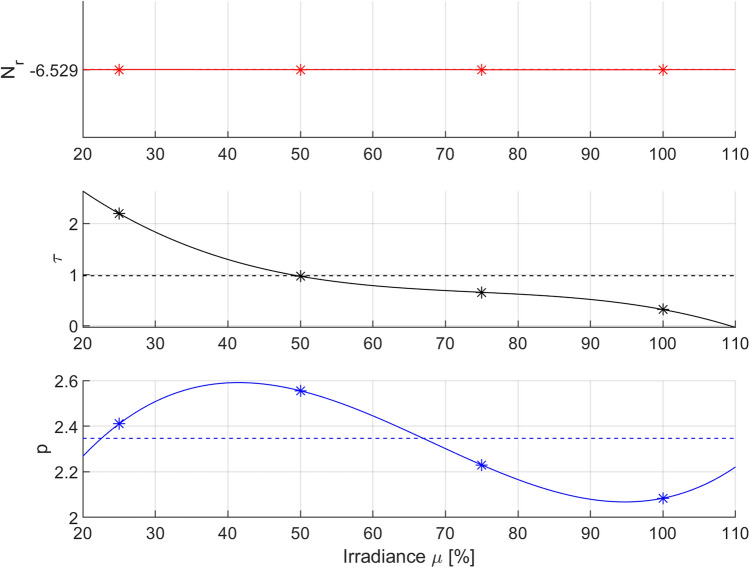
Optimal parameters of the Logistic PDI model (*) for various levels of irradiance *µ* [%] and their polynomial interpolation (–) with RMSE-weighted mean values (–). Optimization was carried out for variation in the *p* and *τ* parameters.

Figure 7 presents the optimal values of parameters *p* and *τ* (with a constant value of parameter *N*_r_) of the Logistic model for the tested irradiation powers and the corresponding approximation curves (third-degree polynomial functions).

The obtained functions that describe the variation in the optimal parameters *p* and *τ* of the Logistic model in the domain of irradiation power are expressed by the following formulas (Eq. 10):

10 $$ \begin{aligned} & N_{rB} = - 6.529 \\ & \tau_{B} \left( \mu \right) = - {0}{\text{.0010}} \times 10^{ - 2} \mu^{3} + {0}{\text{.2233}} \times 10^{ - 2} \mu^{2} - {17}{\text{.2847}} \times 10^{ - 2} \mu + {528}{\text{.0000}} \times 10^{ - 2} \\ & p_{B} \left( \mu \right) = {0}{\text{.0007}} \times 10^{ - 2} \mu^{3} - {0}{\text{.1414}} \times 10^{ - 2} \mu^{2} + {8}{\text{.1593}} \times 10^{ - 2} \mu + {114}{\text{.6000}} \times 10^{ - 2} . \\ \end{aligned} $$

Equations (3) and (10) constitute the next possible version of the extended Logistic PDI model obtained for the case of variation in two selected parameters (*p* and *τ*) of the Logistic model (case B), as indicated by the index B used in the parameter subscript. The total number of model parameters decreased compared to that in case A and was equal to n = 9 in this case, while the mean value of the RMSE quality criterion increased and was equal to 0.2435. However, the increase in the mean value of the RMSE indicator to 0.2435 did not cause significant changes in the quality of fitting of the mortality curves to the experimental data, which can be seen by comparing Fig. 6 (solid lines) with Fig. 4. Thus, this result confirms the hypothesis that the *N*_r_ parameter of the Logistic model can be excluded from the need for variation without a significant reduction in the modeling quality. This is consistent with the assumptions because this parameter is responsible for the residual value of the microbial population and has little effect on the rate of decrease in the mortality curve.

However, setting the *N*_r_ parameter at a constant value requires new calculations to analyze the variation in optimal parameters of the Logistic model. Using formulas (5) and (7), new weighted mean values of the optimal parameters were calculated as follows:

11 $$ \overline{N}_{rB} = - 6.529,\quad \overline{\tau }_{B} = 1.045,\quad \overline{p}_{B} = 2.421. $$

In addition, the following new coefficients of variation were obtained, respectively:

12 $$ CV_{{N_{rB} }} = 0\% ,\quad CV_{\tau B} = 83.90\% ,\quad CV_{pB} = 8.87\% . $$

The mean values of parameters (11) are marked in Fig. 7 with dashed lines, and the coefficients of parameter variation (12) are given in Table 4. As expected, it can be seen that the coefficient of variation of parameter *N*_r_ in this case is equal to zero. It can also be seen that the coefficients of variation of parameters *p* and *τ* have slightly decreased compared with the results from case A; however, parameter *τ* still shows a significantly higher (approximately ten times) sensitivity to variation in irradiation power than parameter *p*. A similar ratio of coefficients of variation of parameters *p* and *τ* was observed in case A. However, it should be clearly noted here that this did not have to happen—in general, setting a chosen parameter to a constant value can significantly affect the mutual ratios of coefficients of variation of other parameters. Therefore, each time after the selected parameter has been varied, it is necessary to reanalyze the variation in the remaining parameters of the identified model.

#### Case C: variation in the *τ* parameter for constant *N*_r_ and *p*

The results of the analysis of parameter variation presented in case B led to the formulation of the hypothesis that omitting the variation in parameter *p* of the Logistic model may also give similar results in terms of the RMSE quality indicator to the results obtained in case A and case B. Therefore, in the next example, it was assumed that only parameter *τ* of the Logistic model was modified, while parameters *N*_r_ and *p* were set to constant values (11) determined in case B. As in the previous case, this required repeating of the parameter optimization procedure for the same experimental data. The optimization results for the *τ* parameter of the Logistic model with constant values for parameters *N*_r_ and *p* are shown in Fig. 6 (dashed lines) and Table 5.

**Table 5:** Results of parameter optimization of the Logistic PDI model with constant *N*_r_ and *p* parameters for various levels of irradiance *µ* [%].

Irradiance	RMSE-optimized parameters			RMSE
	N_ropt_	τ_opt_	p_opt_	
*µ*_1_ = 100%	− 6.529	0.318	2.421	0.2853
*µ*_2_ = 75%		0.653		0.2597
*µ*_3_ = 50%		0.974		0.1664
*µ*_4_ = 25%		2.200		0.3072
RMSE-weighted mean	− 6.529	1.001	2.421	0.2547
Coefficient of variation	0%	82.06%	0%	–

**Figure 8:**
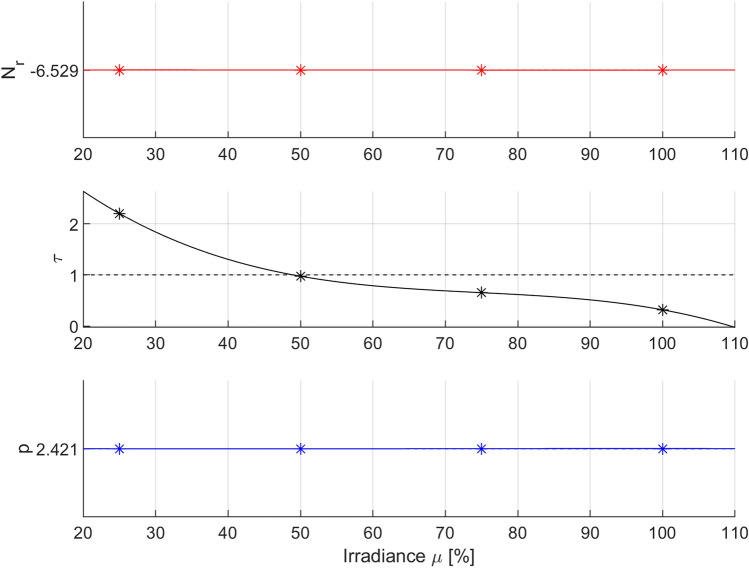
Optimal parameters of the Logistic PDI model (*) for various levels of irradiance *µ* [%] and their polynomial interpolation (–) with RMSE-weighted mean values (–). Optimization was carried out for variation in the *τ* parameter.

Figure 8 presents the optimal values of parameter *τ* (with constant values of parameters *p* and *N*_r_) of the Logistic model for the tested irradiation powers and the corresponding approximation curve (third-degree polynomial function).

The obtained function describing the variation in the optimal value of parameter *τ* of the Logistic model in the domain of irradiation power is expressed as follows (Eq. 13):

13 $$ \begin{aligned} & N_{rC} = - 6.529 \\ & \tau_{C} \left( \mu \right) = - {0}{\text{.0010}} \times 10^{ - 2} \mu^{3} + {0}{\text{.2194}} \times 10^{ - 2} \mu^{2} - {17}{\text{.0733}} \times 10^{ - 2} \mu + {525}{\text{.0000}} \times 10^{ - 2} \\ & p_{C} = 2.421. \\ \end{aligned} $$

Equations (3) and (13) constitute the next possible version of the extended Logistic PDI model obtained for the case of variation in only one selected parameter (τ) of the Logistic model (case C), as indicated by the index C used in the parameter subscripts. The total number of parameters of the Logistic model decreased compared to that in case B and was equal to n = 6 in this case, while the mean value of the RMSE fitting quality criterion increased and was equal to 0.2547. However, as in case B, the deterioration of the RMSE fitting quality indicator was not so significant, which can be seen by comparing solid lines and dashed lines in Fig. 6. Thus, this finding confirms the hypothesis that the *N*_r_ and *p* parameters of the Logistic model can be excluded from the need for variation without a significant deterioration in the modeling quality.

The weighted mean values of the optimal parameters calculated from formula (Eq. 5) in this case are as follows:

14 $$ \overline{N}_{rC} = - 6.529,\quad \overline{\tau }_{C} = 1.001,\quad \overline{p}_{C} = 2.421. $$

The coefficients of variation calculated from formula (Eq. 7) of the individual parameters of the Logistic model are as follows:

15 $$ CV_{{N_{rC} }} = 0\% ,\quad CV_{\tau C} = 82.06\% ,\quad CV_{pC} = 0\% . $$

The mean values of the parameters (14) are marked in Fig. 8 with dashed lines, and the coefficients of parameter variation (15) are given in Table 5. It can be seen that the coefficients of variation of parameters *N*_r_ and *p* were equal to zero, while the coefficient of variation of parameter *τ* maintained its value at a similar level as that in case B. The relatively high value of the coefficient of variation of parameter *τ* suggests that the variation in this parameter may be of key importance for maintaining the required quality of model fitting to the experimental data. To confirm this hypothesis, case D was analyzed, in which instead of variation in parameter *τ*, parameter *p* was varied for constant values of parameters *N*_r_ and *τ.*

#### Case D: Variation in the *p* parameter for constant *N*_r_ and *τ*

**Figure 9:**
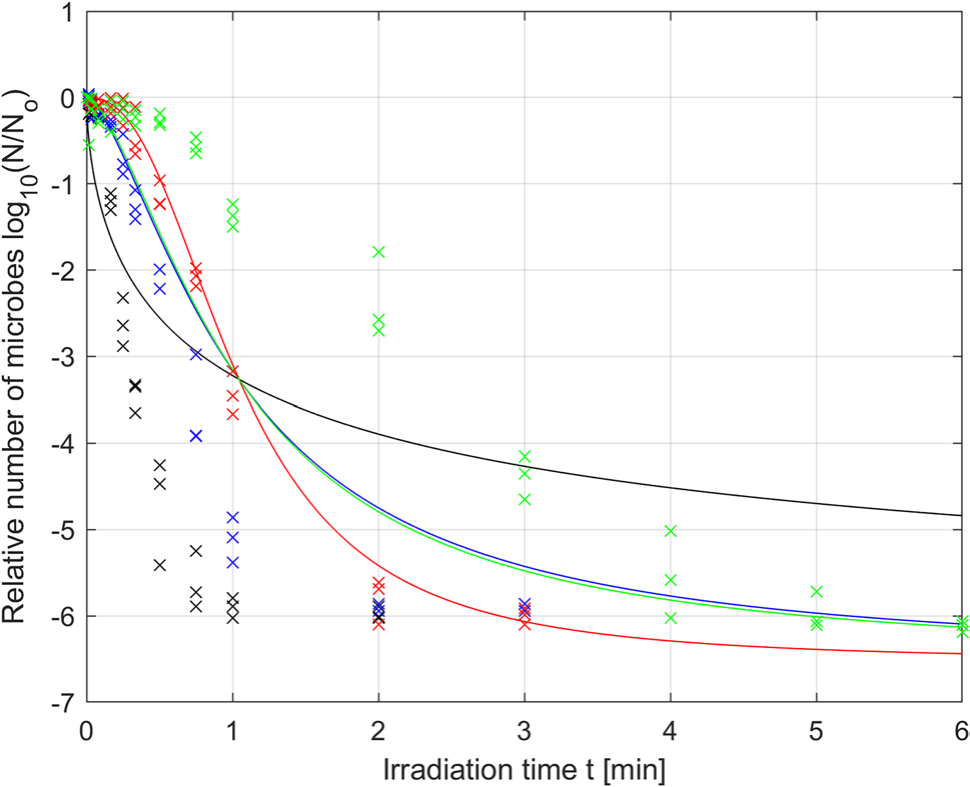
Photodynamic inactivation of *S. agalactiae* for various levels of irradiance: *µ*_1_ = 70 mW/cm^2^ (black), *µ*_2_ = 52.5 mW/cm^2^ (blue), *µ*_3_ = 35 mW/cm^2^ (red), *µ*_4_ = 17.5 mW/cm^2^ (green): experimental data (x) and microbial survival curves (–) of the Logistic PDI model optimized for variation in only the *p* parameter.

In this case, it was assumed that only parameter *p* of the Logistic model will be modified, while parameters *N*_r_ and *τ* will be set to constant values (11) determined in case B. The optimization results of the *p* parameter of the Logistic model for constant values of the *N*_r_ and *τ* parameters are shown in Fig. 9 and Table 6.

**Table 6:** Results of parameter optimization of the Logistic PDI model with constant *N*_r_ and *τ* parameters for various levels of irradiance *µ* [%].

Irradiance	RMSE-optimized parameters			RMSE
	N_ropt_	τ_opt_	p_opt_	
*µ*_1_ = 100%	− 6.529	1.045	0.602	1.5812
*µ*_2_ = 75%			1.551	0.7858
*µ*_3_ = 50%			2.439	0.2104
*µ*_4_ = 25%			1.565	1.0631
RMSE-weighted mean	− 6.529	1.045	2.029	0.9101
Coefficient of variation	0%	0%	46.31%	−

As seen in Table 6, in this case, the mean value of the RMSE fitting quality criterion is significantly higher compared to that in case C and is equal to 0.9101. It can be clearly seen in Fig. 9 that the quality of fitting the Logistic model to the experimental data obtained by changing parameter *p* for constant values of parameters *N*_r_ and *τ* does not give the expected results.

#### Case E: Logistic PDI model with all constant parameters: *N*_r_, *p* and *τ*

**Figure 10:**
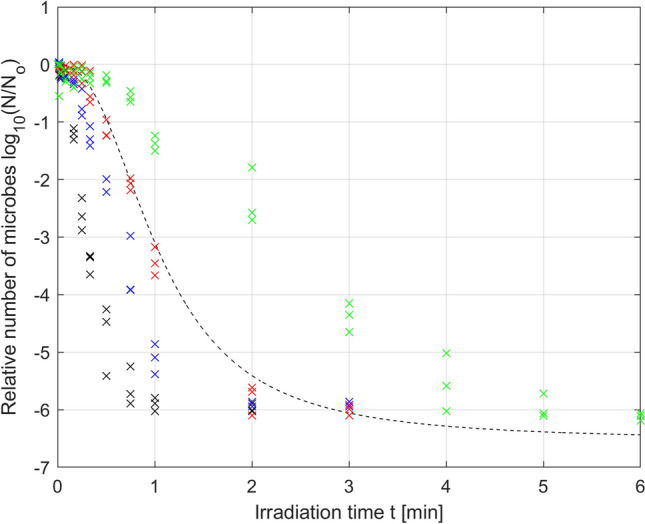
Photodynamic inactivation of *S. agalactiae* for various levels of irradiance: *µ*_1_ = 70 mW/cm^2^ (black), *µ*_2_ = 52.5 mW/cm^2^ (blue), *µ*_3_ = 35 mW/cm^2^ (red), *µ*_4_ = 17.5 mW/cm^2^ (green): experimental data (x) and microbial survival curve (–) of the Logistic PDI model optimized for all constant parameters.

The last considered case is the variant in which all parameters of the Logistic model are excluded from modification. In this case, it was assumed that all parameters of the Logistic model will be set to constant values (11) determined in case B. The optimization results in this case are shown in Fig. 10 and Table 7.

**Table 7:** Results of parameter optimization of the Logistic PDI model with all constant parameters for various levels of irradiance *µ* [%].

Irradiance	RMSE-optimized parameters			RMSE
	N_ropt_	τ_opt_	p_opt_	
*µ*_1_ = 100%	− 6.529	1.045	2.421	2.1824
*µ*_2_ = 75%				0.9013
*µ*_3_ = 50%				0.2105
*µ*_4_ = 25%				1.1300
RMSE-weighted mean	− 6.529	1.045	2.421	1.1060
Coefficient of variation	0%	0%	0%	–

Of course, in this case, the quality of model fitting significantly deteriorates compared to all previous cases. This confirms the need to modify the parameters of the Logistic model.

## Discussion

The problem of mathematical inclusion of the effect of changes in irradiance into the PDI models is not a trivial issue. The discussed cases show that for multiparameter PDI models, there are many variants of parameter modification that take into account the utilized irradiance. The parameter dependence on irradiation power was adopted as the method of parameter modification in this study. An appropriate dependence on irradiation power was obtained by approximation of parameter variation by third-degree polynomial functions within the tested irradiation power range. That choice was sufficient in each of the considered cases. The model fitting quality and the degree of complexity of the proposed parameter modification were the most important comparative criteria utilized in this analysis. However, the fulfillment of both of these criteria usually results in mutual contradiction. In general, the decrease in the number of parameters to be modified leads to deterioration in the fitting quality indicators. Deterioration of the model fitting quality may also be caused by inappropriate selection of the function that approximates the optimal parameter variations. Each of the considered cases is characterized by a different fitting quality and a different degree of model complexity related to the total number of parameters. The most important results of this study are presented in Table 8.

**Table 8:** Comparative analysis of several case studies of parameter modification of the Logistic PDI model for various irradiances *µ* [%].

Modification case	Parameters of extended logistic PDI model	Total number of parameters	Mean RMSE
A	$$\begin{aligned} & N_{rA} \left( \mu \right) = {0}{\text{.0009}} \times 10^{ - 2} \mu^{3} - {0}{\text{.2161}} \times 10^{ - 2} \mu^{2} + {17}{\text{.2507}} \times 10^{ - 2} \mu - {1076}{\text{.8000}} \times 10^{ - 2} \\ & \tau_{A} \left( \mu \right) = - {0}{\text{.0015}} \times 10^{ - 2} \mu^{3} + {0}{\text{.3279}} \times 10^{ - 2} \mu^{2} - {25}{\text{.0780}} \times 10^{ - 2} \mu + {710}{\text{.7000}} \times 10^{ - 2} \\ & p_{A} \left( \mu \right) = {0}{\text{.0011}} \times 10^{ - 2} \mu^{3} - {0}{\text{.2330}} \times 10^{ - 2} \mu^{2} + {15}{\text{.6713}} \times 10^{ - 2} \mu - {71}{\text{.2000}} \times 10^{ - 2} \\ \end{aligned}$$	12	0.2207
B	$$\begin{aligned} & N_{rB} = - 6.529 \\ & \tau_{B} \left( \mu \right) = - 0.0010 \times 10^{ - 2} \mu^{3} + 0.2233 \times 10^{ - 2} \mu^{2} - 17.2847 \times 10^{ - 2} \mu + 528.0000 \times 10^{ - 2} \\ & p_{B} \left( \mu \right) = 0.0007 \times 10^{ - 2} \mu^{3} - 0.1414 \times 10^{ - 2} \mu^{2} + 8.1593 \times 10^{ - 2} \mu + 114.6000 \times 10^{ - 2} \\ \end{aligned}$$	9	0.2435
C	$$\begin{aligned} & N_{rC} = - 6.529 \\ & \tau_{C} \left( \mu \right) = - 0.0010 \times 10^{ - 2} \mu^{3} + 0.2194 \times 10^{ - 2} \mu^{2} - 17.0733 \times 10^{ - 2} \mu + 525.0000 \times 10^{ - 2} \\ & p_{C} = 2.421 \\ \end{aligned}$$	6	0.2547
D	$$\begin{aligned} & N_{rD} = - 6.529 \\ & \tau_{D} = 1.045 \\ & p_{D} \left( \mu \right) = {0}{\text{.0018}} \times 10^{ - 2} \mu^{3} - {0}{\text{.4131}} \times 10^{ - 2} \mu^{2} + {26}{\text{.5420}} \times 10^{ - 2} \mu - {277}{\text{.2000}} \times 10^{ - 2} \\ \end{aligned}$$	6	0.9101
E	$$\begin{aligned} & N_{rE} = - 6.529 \\ & \tau_{E} = 1.045 \\ & p_{E} = 2.421 \\ \end{aligned}$$	3	1.1060

The purpose of this comparative analysis is to find an effective compromise between the fitting quality and the total number of parameters of the proposed extended PDI model (Eq. 3) in the whole tested irradiation power range. In case A, all parameters of the Logistic PDI model were modified. This solution provides the best fit results but has a serious drawback in terms of model complexity. In this case, the total number of parameters is equal to 12, which makes it very difficult to use such a model. In case B, where only two parameters were modified, the total number of parameters decreased compared to case A and equaled 9, but at the cost of model fitting quality. However, this difference is not significant. In case C, the number of modified parameters was reduced to 1, and the total number of extended model parameters was reduced to 6. As in case B, the model fitting quality slightly deteriorated, but this difference was still not significant. In case D, the number of modified parameters was also reduced to 1, with the total number of extended model parameters also equal to 6, but the model fitting quality deteriorated significantly. Case E, in which no parameters were modified, exhibited the worst model fitting quality.

A comparative analysis of cases C and D confirms the hypothesis in case C that the variation in the *τ* parameter is of key importance for maintaining a high quality of the extended Logistic model within the tested irradiation power range. This may be slightly surprising because the combination of the *p* and *τ* parameters is responsible for the shape and the rate of decrease of the microbial mortality curve in the Logistic model, so it would seem that both these parameters should be changed to maintain the required fitting quality. However, the results of the conducted analysis of parameter variation clearly show that the variation in parameter *p* in the case under study can be omitted. Under the studied conditions, the variation in the irradiation power most likely affects such characteristics of the mortality curve for which the *τ* parameter of the Logistic model is decisively responsible. The only problem is finding the appropriate values of parameters *N*_r_ and *p*, for which the variation in parameter *τ* gives satisfactory results for fitting the model to the experimental data. The presented method of parameter modification describes the method for finding these values by using the coefficients of variation of the Logistic model parameters.

The conducted comparative analysis shows that the most favorable of the presented cases of parameter variation is case C. In this case, the best compromise between the total number of model parameters (n = 6) and the mean RMSE fitting indicator (0.2547) were obtained. The final extended Logistic PDI model that takes into account the effect of changes in the irradiance can be presented in the following form (Eq. 16):

16 $$ \log \left( {\frac{N\left( t \right)}{{N_{o} }}} \right) = \overline{N}_{rC} \left( {1 - \frac{1}{{1 + \left( {\frac{t}{{a_{\tau 3} \mu^{3} + a_{\tau 2} \mu^{2} + a_{\tau 1} \mu + a_{\tau 0} }}} \right)^{{\overline{p}_{C} }} }}} \right) $$

where the parameters of this model are as follows:

17 $$ \begin{aligned} & \overline{N}_{rC} = - 6.529, \\ & \overline{p}_{C} = 2.421, \\ \end{aligned} $$

while the *τ* parameter is expressed as a four-element vector of polynomial coefficients:

18 $$ {{\varvec{\uptau}}} = \left[ {\begin{array}{*{20}c} {{\text{a}}_{\tau 3} } & {{\text{a}}_{\tau 2} } & {{\text{a}}_{\tau 1} } & {{\text{a}}_{\tau 0} } \\ \end{array} } \right]^{T} = 10^{ - 2} \times \left[ {\begin{array}{*{20}c} {{0}{\text{.0010}}} & {{0}{\text{.2194}}} & { - {17}{\text{.0733}}} & {{525}{\text{.0000}}} \\ \end{array} } \right]_{.}^{T} $$

**Figure 11:**
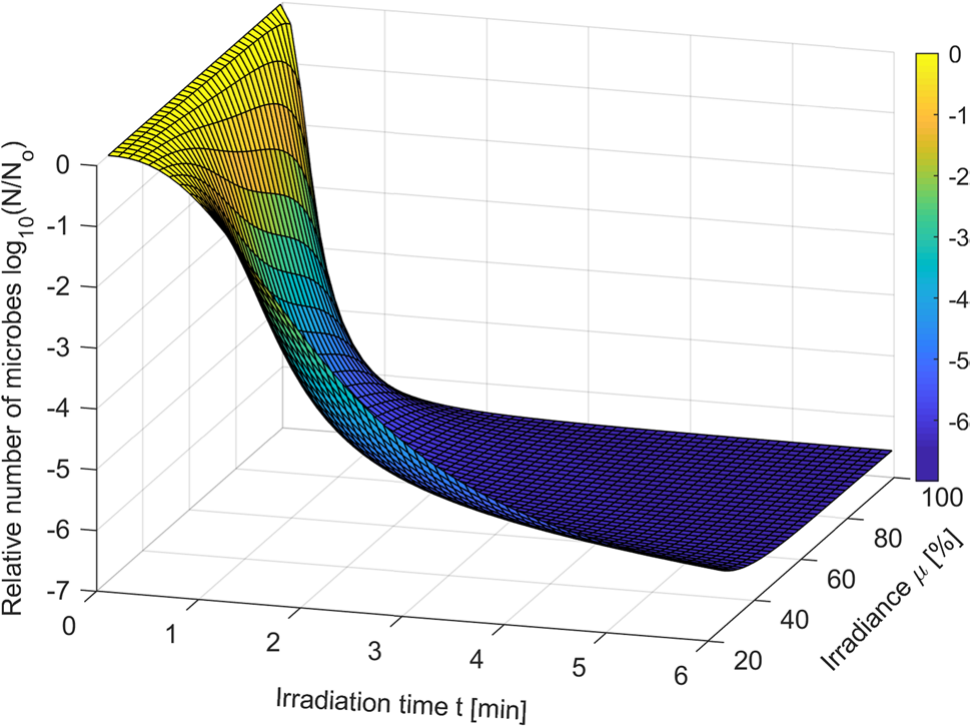
Extended logistic model of *S. agalactiae* photodynamic inactivation in the whole tested range of irradiance *µ* [%].

The extended Logistic PDI model (Eqs. 16–18) obtained in the conducted research effectively describes the dynamics of microorganism mortality in the whole tested irradiation power range *µ* [%]. The usefulness of this model is illustrated in Fig. 11.

The resulting extended model expressed in the form (Eqs. 16–18) can be successfully used to estimate the required irradiation time for any power level within the tested range. Assuming *x*_req_ = log(N/No) as the relative logarithmic number of microorganisms required and *µ* [%] as the level of irradiance used, the required irradiation time *t*_req_ [min] can be calculated from the following formula (Eq. 19):

19 $$ t_{req} \left( {x_{req} ,\mu } \right) = \left( {\frac{1}{{1 - \frac{{x_{req} }}{{\overline{N}_{rC} }}}} - 1} \right)^{{\frac{1}{{\overline{p}_{C} }}}} \cdot \left( {{\text{a}}_{\tau 3} \mu^{3} {\text{ } + \text{ a}}_{\tau 2} \mu^{2} + {\text{a}}_{\tau 1} \mu + {\text{a}}_{\tau 0} } \right) $$

where all parameters are the same as in Eqs. (17, 18).

**Figure 12:**
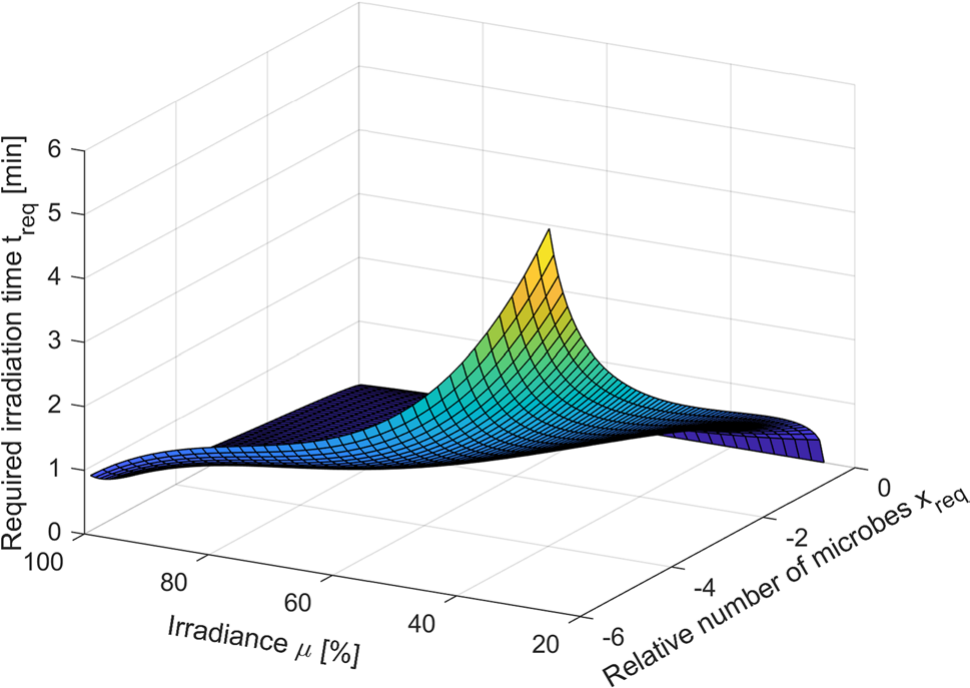
*S. agalactiae* PDI model estimating the required irradiation time *t*_req_ [min] for required size of microorganism population *x*_req_ and used level of irradiance *µ* [%].

The mathematical expression presented in Eq. (19) is a model describing the dependence of the required irradiation time *t*_req_ [min] as a function of the required size of the microorganism population *x*_req_ = log(N/No) and used level of irradiance *µ* [%]. The usefulness of this model is shown in Fig. 12.

**Figure 13:**
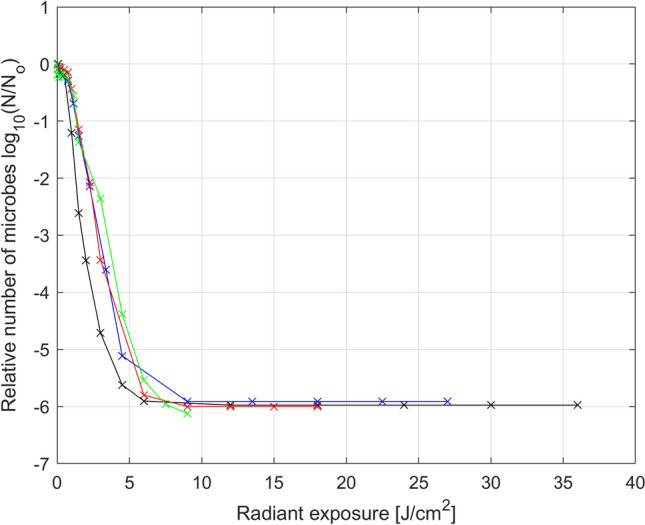
Photodynamic inactivation of *S. agalactiae* for various radiant exposures (light doses) and various levels of irradiance: *µ*_1_ = 70 mW/cm^2^ (black), *µ*_2_ = 52.5 mW/cm^2^ (blue), *µ*_3_ = 35 mW/cm^2^ (red), *µ*_4_ = 17.5 mW/cm^2^ (green): averaged experimental data (x) and its linear interpolation (–).

Analysis of the number of microbes as dependent of radiant exposure (light dose) can be an added value to the resulting extended model (Eq. 19). The results presented in Fig. 13 show that the greater the radiant exposure is, the smaller the population size, but for various levels of irradiance the curves have a slightly different shape and differ for lower radiant exposures (less than 10 J/cm^2^). In particular, the curve for irradiance *µ*_1_ = 70 mW/cm^2^ is slightly deviated from the others. This information can be helpful to determine the required irradiation time in relation to level of irradiance in PDI processes. The obtained results lead to the conclusion that in case of the current study the use of higher irradiance can be beneficial to minimize the radiant exposure. Nevertheless, one must be aware that irradiance is closely related to temperature; thus, this parameter should be adjusted in such manner to avoid bacterial killing from heat.

The results of the mathematical analysis made in the current study differ significantly from the results available in the literature on the subject (in particular, in the works of the Luksiene’s team)^13–15,17^. In the studies by Luksiene’s group, only the results of matching the classic 3-parameter Logistic model depending on different incubation times are shown (which, like the exposure power, is one of the factors affecting the inactivation process). However, the presented results do not formulate any mathematical sub-model, which would approximate the obtained relationships to include them in the classic model. These reports do not discuss any way to modify the parameters of the classic model to optimize its extension. There is also no analysis of the significance of individual model parameters due to the examined factor, or any other method that could be used while minimizing the number of additional model parameters that are required to model the obtained relationships. Ultimately, there are also no inverse models that allow predicting the minimum necessary exposure time depending on the factor tested. Our analysis evidence which of the parameters of the classic 3-parameter Logistic model is the most important one due to the exposure power factor. The fact that it was possible to select such a parameter of the studied model is also a remarkable achievement. In the case of multi-parameter models, this problem is not a trivial matter. In general, such unambiguous selection of a parameter responsible for specific model properties may be difficult or even impossible to obtain. Then, any possible modifications to the model would have to apply to all its parameters, which would usually cause a sudden increase in the number of additional parameters and a significant and disproportionate increase in modeling costs. In addition, thanks to the conducted research, it was possible to formulate an inverse model (Eq. 19, Fig. 12) that allows to predict the minimum necessary irradiation time needed to achieve the required level of population size for the adopted exposure power. Obtaining such a model could be considered the main motivation for extending the classic model.

## Final conclusions

One of the methods of taking into account the effect of irradiance in the identified PDI model may be to make its parameter appropriately dependent on that factor. Unfortunately, for multiparameter PDI models, an appropriate variation in all model parameters can lead to excessive complications and an increase in the number of parameters. Therefore, striving to simplify the modifications included in the model is warranted. Determining which model parameters need to be varied and which do not is not a trivial matter in general. As shown in the manuscript, by properly conducting an analysis of model parameter variation, it is possible to effectively limit the total number of parameters of the examined model without worsening the model fitting quality to the collected experimental data. The main result of the manuscript is the extended Logistic PDI model that takes into account the irradiance by varying the appropriately selected parameters of this model with functions depending on the used irradiation power. Furthermore, the developed extended Logistic PDI model can be successfully used to estimate the required irradiation time for any power level within the tested range.

## Data Availability

The datasets generated during and/or analysed during the current study are available from the corresponding author on reasonable request.
